# A rapid density method for taxi passengers hot spot recognition and visualization based on DBSCAN^+^

**DOI:** 10.1038/s41598-021-88822-3

**Published:** 2021-05-03

**Authors:** Zihe Huang, Shangbing Gao, Chuangxin Cai, Hao Zheng, Zhigeng Pan, Wenting Li

**Affiliations:** 1 Faculty of Computer and Software Engineering, Huaiyin Institute of Technology, Huaian, 223003 People’s Republic of China; 2 Key Laboratory of Intelligent Information Processing, Nanjing Xiaozhuang University, Nanjing, 211171 People’s Republic of China; 3 Institute of VR and Intelligent System, Hangzhou Normal University, Hangzhou, 311121 People’s Republic of China

**Keywords:** Computer science, Scientific data, Software

## Abstract

With the development of city size and vehicle interconnection, visual analysis technology is playing a very important role in the course of city calculation and city perception. A Reasonable visual model can effectively present the feature of city. In order to solve the problem of traditional density algorithm that cluster the large scale data slowly and cannot find cluster centers to adapt taxi track data. The DBSCAN^+^ (density-based spatial clustering of applications with noise plus) algorithm that can split data and extract maximum density clusters under the large scale data was proposed in the paper. The passenger points should be cleaned from the original point of the passenger trajectory data firstly, and then the massive passenger points are sliced and clustered cyclically. In the clustering process, the cluster centers can be extracted based on maximum density, and finally the clustering results are visualized according to the results. The experimental results show that compared with other popular methods, the proposed method has significant advantages in clustering speed, precision and visualization for large-scale city passenger hotspots. Moreover, it provides important decisions for further urban planning and promotes the traffic efficiency.

## Introduction

With the development of urban traffic, visual analysis technology plays an important role in the analysis process of urban traffic hot spots. Taxi is not available during peak hours, and usage during off-peak hours is low. This taxi imbalance problem can be resolved by analyzing the spatial data and predicting the demand hotspots to identify areas with potential passengers^1,2^. The discovery of hot spots can bring tremendous benefits to drivers and passengers^3,4^. Luo et al. put forward a visual analysis method for urban roads^5^. More cities have established taxi stands to advocate and to guide passengers to hail a taxi. However, most of taxi stands have low rate of usage^6^. Building a reasonable visual model can effectively display the spatial and temporal distribution characteristics of urban hot spots. The formation of hotspots is strongly correlated with many features, i.e., time, space, and the distribution of points of interest^7^. Detailed urban land social function identification is an integral part of urban planning^8^. It also can provide important decision information for urban planning and so on.

DBSCAN is the most popular clustering method based on density. Kumar et al. proposed a fast DBSCAN clustering algorithm^9^ which uses group method to accelerate neighborhood search. The traditional DBSCAN algorithm has some limitations in clustering mass taxi traffic track data points, such as being unable to adapt to large-scale data, unable to identify cluster centers, and slow and single thread clustering speed process.

Among the existing techniques, there are five ways to visualize taxi hot spots based on DBSCAN traffic data:

1. Icon-based visualization: The GBADBSCAN algorithm^10^ used icons to represent passenger hotspots in data visualization.
2. Color features visualization: For example, the LCS-BASED DBSCAN clustering algorithm^11^ used different colors to distinguish in the visualization of passenger hot spots.
3. Time-axis features visualization: For example, Zhao et al. proposed a time-axis based passenger hot spot area^12^, and used different colors to mark the passenger hot spot area in different time periods.
4. By dividing grid cells: DBSCAN algorithm based on network constraints^13^ and Zheng^14^ proposed a grid-based k-means traffic hot area recognition algorithm, while Zhou et al. proposed a clustering model^15^ for detecting track points using the potential threshold method of data field to extract hot spots, Kong et al.^16^ filled different areas with different colors to display the interactive intensity of this target area, showing the number of taxi trips from different areas (grid) to major hospitals in Beijing. Wang R segments road network into several road clusters and use ranking-based extreme learning machine (ELM) model to evaluate the passenger-finding potential of each road cluster^17^.
5. By processing image: Liu D et al. proposed a solution that projects all points to a density image and execute variant of the DPC algorithm on the processed image^18^.

These algorithms improved DBSCAN, whether based on icon-based visualization, color feature visualization, time-axis feature visualization or grid-based partitioning, all achieve certain clustering results in the cluster hotspot region and match the clustering results on the map in a certain way. However, the existing DBSCAN improved algorithm is not efficient in processing big data, and the clustering accuracy is insufficient. In the visualization technology, hot spots or sections are not displayed on the map according to the thermal size, and the thermal relationship between hot spots cannot be judged intuitively.

The DBSCAN^+^ based taxi passenger hot spot visualization method is proposed in this paper. Compared with existing technologies, cluster centers are introduced into the traditional DBSCAN algorithm to identify cluster centers. Parallel computing and fractal dimension reduction processing can adapt to large-scale data and cluster quickly. It overcomes the problem that the existing technology cannot adapt to mass data and lack of precision in the extraction of hot spots for taxi passengers^19^. In terms of visualization effect, the method presented in this paper can directly and effectively show the passenger carrying heat capacity of each region of the city. The actual geographic location table of passenger hot spots after the heat output is refined. It overcomes the problem that mass clustering hot spots are not visualized directly on the map and are not easy to make decisions. Then, relevant departments can make decisions on taxi operation and dispatch according to the degree of aggregation in each hot area and help taxi drivers to find hot spots and wait for passengers more quickly. It is also great significance to optimize the spatial allocation of public facilities and rationally allocate public resources to alleviate traffic pressure. In addition, taxi passenger hot–spots recognition can also contribute to identify a location in each cluster as a candidate bus taxi stands and bus stop or address the problem of night-bus stop planning by investigating the characteristics of taxi GPS trajectories and transactions^20–22^. The trajectory data provides us with a unique new perspective to discover and understand human behavior patterns and potential intelligence in various situations and determine the functions of the hot-spot regions^23,24^.

## Method

**Figure 1:**
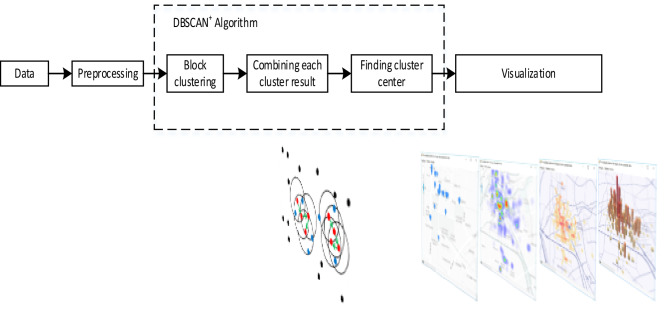
The flow chart of the algorithm.

The algorithm mainly includes Data preprocess, DBSCAN^+^ Algorithm and Visualization. The overall architecture of the algorithm is shown in Fig. 1.

### Preprocessing of trajectory data

Due to the large amount of trajectory data and the influence of GPS device precision and environment and other factors, some abnormal points^25^ were included in the initial GPS data points, which would directly affect the accuracy of subsequent trajectory processing. We firstly preprocessed the data to a certain extent and then clustering it.

#### Trajectory data description

In the taxi GPS data trajectory resources of Huai’an city from 2017 to 2018, the method of removing the interference data from the large initial data is as follows: set the maximum taxi speed as *V*_max_. GPS trajectory data points are extracted from the GPS data trajectory for a period of time, which is denoted as GPS trajectory sequence {*p*_1_, *p*_2_, …*p*_*k*_}, *k* is the number of trace points in the sequence. The reservation is selected to meet the following GPS data trajectory points:

1 $$ 0 < \frac{{distance\left( {p_{i + 1} ,p_{i} } \right)}}{{\left( {t_{{p_{i + 1} }} - t_{{p_{i} }} } \right)}} < V_{\max } $$

where distance (*p*_*i*+1_, *p*_*i*_) represents the distance of the surface of the earth from *p*_*i*+1_ to *p*_*i*_, $t_{{p_{i} }}$ represents the time to collect the *p*_*i*_ track point, and $\left( {t_{{p_{i + 1} }} - t_{{p_{i} }} } \right)$ represents the time difference between point *p*_*i*+1_ and point *p*_*i*_. If *p*_*i*_ does not satisfy the above equation, *p*_*i*_ is a jump point, and the GPS data track point is removed.

The data used in this paper is the taxi GPS trajectory data of Huai’an city from 2017 to 2018. The data collected the taxi trajectory with GPS device in Huai’an city for nearly one and a half years. The data size is about 200G, and the sampling time interval is 30–60 s. Each trajectory point contains the following parameters as Table 1.

**Table 1:** OBD partial data parameter description.

Data field	Data description
Vid	The unique license plate number of a car
Gps_time	The sampling time of sampling point, the representing form is ’yyyy-/MM/-dd HH:mm:ss’
Lon	Longitude (*1,000,000), accurate to 6 decimal places
Lat	Latitude (*1,000,000), accurate to 6 decimal places
Speed	Speed: 0.1 km/h
Mile	Mileage: 0.1 km
Dir	Direction: 0–359, true north is 0, clockwise
Operstate	The passenger status of the taxi at the current sampling point
ClusterId	The cluster ordinal number (0: unclassified, − 1: noise point, positive number: ordinal number)

#### Pick-up and drop-off points

**Figure 2:**
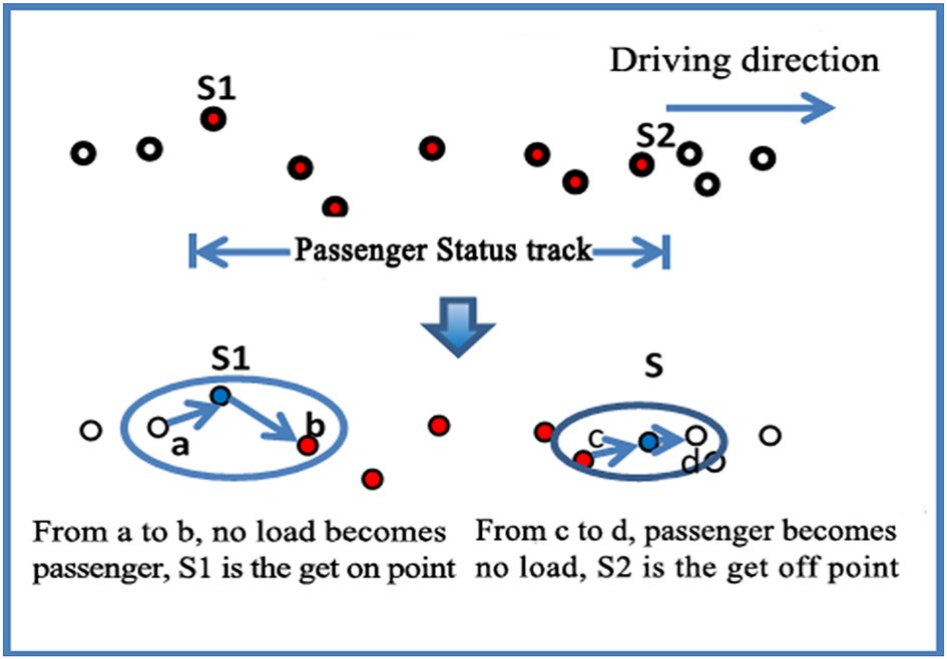
The change status of passengers at data points of getting on and off the bus.

In order to facilitate the extraction of data points from the bus, this paper uses cursor operation to traverse all data in the database. The data point of boarding, that is, the vehicle *operstate* changes from no-load state to passenger state at this moment, while the data point of getting off is opposite. Therefore, the operation status of taxi data collection is selected as the judgment basis, and the passenger status of the two tracking points before and after comparison is used to determine whether it is the data point of getting on and off the taxi, as shown in Fig. 2.

### DBSCAN^+^ algorithm

The traditional DBSCAN algorithm has the limitations of not being able to adapt to large-scale data, not being able to identify cluster centers, and slow single-threaded clustering speed in the process. In this section, the practical application of DBSCAN algorithm to cluster mass trajectory points is presented, and the DBSCAN^+^ algorithm is proposed for the limitation. Finally, experimental data were compared from the three aspects of clustering accuracy, algorithm complexity and resource utilization, indicating the superiority of DBSCAN^+^ algorithm in clustering massive trajectory points.

#### DBSCAN algorithm

Based on density clustering algorithms, the primary goal is to look for dense areas separated by low-density areas. Different from the density based clustering algorithm, the distance based clustering algorithm's clustering results are spherical clusters, while the density based clustering algorithm can find clusters of any shape, which has a good effect on the processing of data with noise points. DBSCAN is the most commonly used density—based clustering method. The algorithm divides data points into the following three types:

- Emphasis: It contains a point in the radius *Eps* that exceeds the number of *MinPts*.
- Boundary point: It contains less than *MinPts* within a radius of a share, and it's in the neighborhood of the nuclear core.
- Noise point: It's neither a core point nor a boundary point.

**Figure 3:**
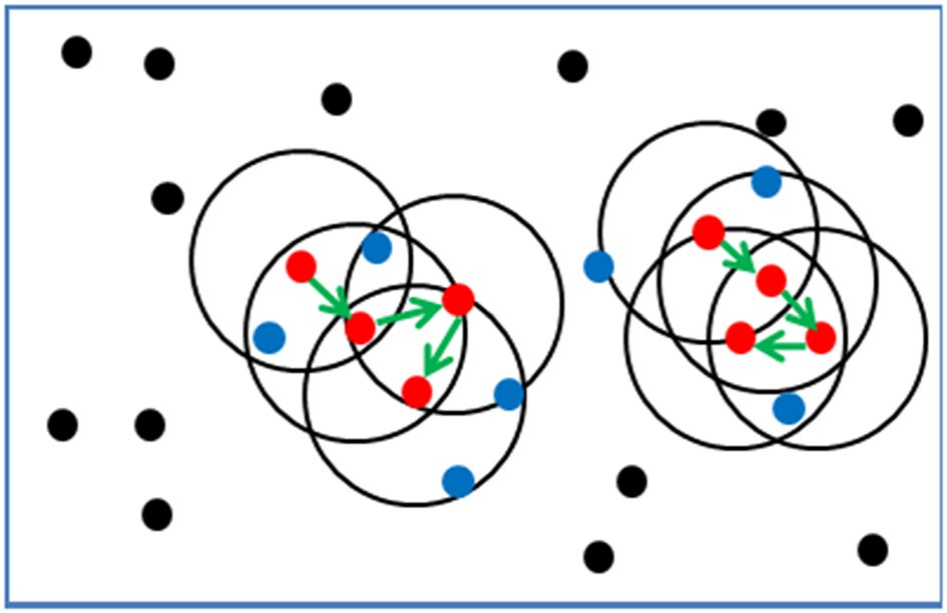
DBSCAN clustering results.

The basic principle of DBSCAN algorithm is to find the maximum density associated in the data point set by setting the scanning radius *Eps* and the minimum inclusion point *MinPts*. As shown in Fig. 3, the figure is the DBSCAN clustering result graph with *MinPts* = 5, *Eps* = 1 as the parameter, where the red point is the core point, the blue point is the boundary point and the black point is the noise point.

Therefore, the algorithm simply considered the distance between data points and the minimum number of inclusion points required for each core point. In the case of a large amount of data, many problems occurs frequently, such as excessive trace points, wide coverage area, poor visualization effect and inability to find hot spots accurately often occur in clusters.

#### DBSCAN^+^ algorithm

The core idea of DBSCAN^+^ algorithm is to find the first cluster, then find the cluster centers of each cluster in the clustering results and cluster the results again, so as to effectively reduce the algorithm's time and space complexity. At the same time, through clustering algorithm based on density determine the center of each cluster^26^, thus locating passenger hotspots accurately. The method to determine the cluster center mentioned in the article is to determine the geometric center. This algorithm is more scientific based on the density center.

The algorithm first separates the noise point and core point *P* based on *Eps* and *MinPts*, and the neighbor node set *N*{*N*_1_, *N*_2_, *N*_3_…} of core point *P* is obtained. Then the core *P* point is extended cluster operation.

Extension cluster: The neighbor points of the core points were divided into clusters, and the neighbor core points *N*_*i*_ and its neighborhoods were added to the cluster. The following is the definition of maximum density connection after extended cluster:

2 $$ N = \left\{ {\bigcup\limits_{i = 1}^{n} {X_{i} } \bigcup {P\bigcup\limits_{j = 1}^{n} {X_{j} } } \in N_{i} domain\left( {N_{i,count} > Minpts} \right)} \right\} $$

where *N* represents the extended cluster set of the maximum density connected, *P* represents the core point of the cluster, and *X*_*i*_ represents the neighbor node set of the cluster, *X*_*i*_ represents the neighborhood set of the neighbor node in the cluster that is larger than the minimum number of cluster points.

#### Determining the cluster center

As the upper and lower passenger points of taxis are generally distributed on both sides of the road, the clustering results tend to be a banded cluster. The cluster center simply takes the center of mass of the cluster as the cluster center, so it is easy to fail to accurately reflect the passenger hot spots. Therefore, the density based method is adopted to determine the cluster center.

Recognition of class clusters is a prerequisite for the operation of cluster center recognition. The cluster centers of class are identified by the following formula:

3 $$\updelta _{k} = \max \left( {\rho_{{k_{i} }} } \right) $$

4 $$ \rho_{{k_{i} }} = \frac{{Neighbours\left( {} \right)}}{count\left( N \right)} \times 100\% $$

In the formula, δ_*k*_ represents the cluster centers of class clusters, $\rho_{{k_{i} }}$ represents the density of point *N*_*i*_ in the cluster, the function of *Neighbours*() represents the number of neighboring nodes, and *count*() represents the total number of points in the cluster represents the cluster centers of class clusters, $\rho_{{k_{i} }}$ represents the density of point *N*_*i*_ in the cluster, *Neighbours*() presents the number of neighboring nodes, and *count*() represents the total number of points in the cluster.

**Figure 4:**
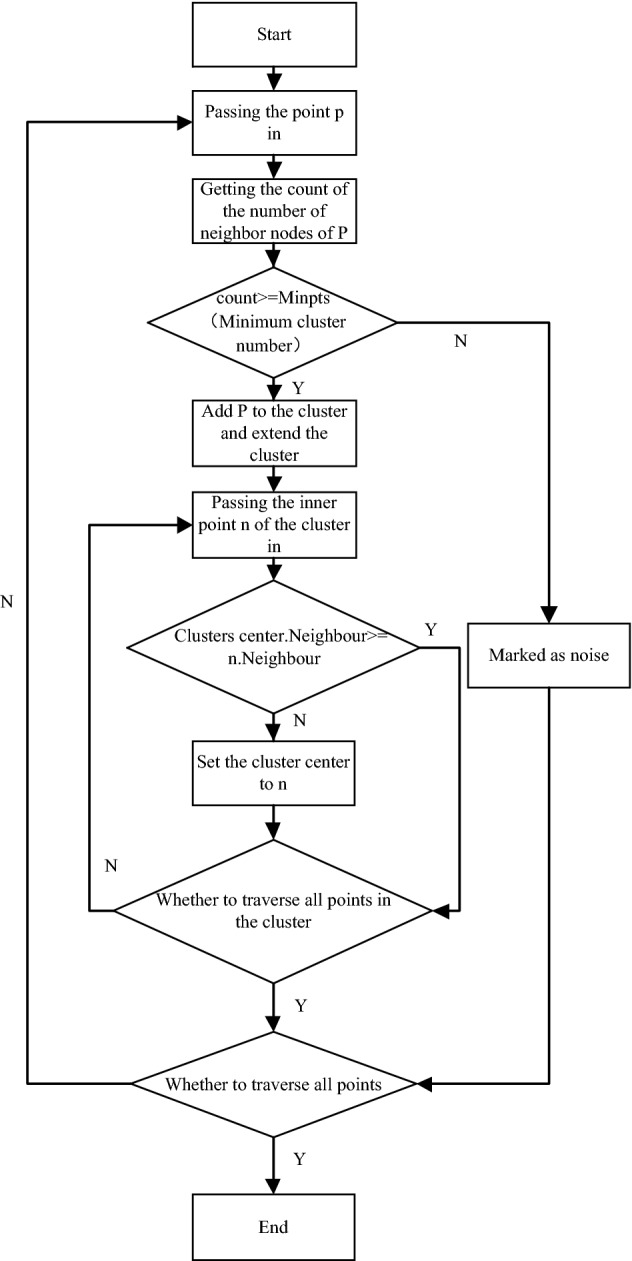
The cluster center identification flowchart.

The algorithm is based on the density cluster center recognition method, and its principle is to record the number of points in the neighborhood point set *N* of nodes in each cluster when obtaining the operation of class cluster. The density is calculated for each point in the cluster. The calculation method is to calculate the ratio of the number of neighbor points at the point *P* to the total number of points in the cluster, which is denoted as the density of *P* points. Finally, we find out the point with the highest density in the cluster which contains the largest number of neighbor nodes as the center of the cluster. The cluster center identification flowchart is shown in Fig. 4.

Dijkstra-based dynamic time warping distance measure, which is suitable for large numbers of overlapping trajectories in dense road networks^27^. DBSCAN^+^ uses the longitude and latitude to calculate the spherical distance. The algorithm determined the distance between two data points according to the longitude and latitude information of two points. However, the surface of the earth is curved, so when calculating the distance between two geographical positions, we cannot use the simple Euclidean distance, we need to consider the actual surface distance:

5 $$ a = lat_{1} - lat_{2} $$

6 $$ b = lon_{1} - lon_{2} $$

7 $$ L = 2*R*\sin^{ - 2} \sqrt {\left( {\sin \frac{a}{2}} \right)^{2} + \cos \left( {lat_{1} } \right)*\cos \left( {lat_{2} } \right)\left( {\sin \frac{b}{2}} \right)^{2} } $$

where *lat*_1_ represents the latitude of the first locus, *lat*_2_ represents the latitude of the second locus, *lon*_1_ represents the longitude of the first locus, *lon*_2_ represents the longitude of the second locus, and R represents the radius of the earth. The formula error is less than 0.2 m.

According to the algorithm, users can effectively find the maximum density point in each cluster. The method of using cluster center instead of cluster class can not only make the output more concise, but also make the hot spot display more accurate.

#### Partitioned clustering

**Figure 5:**
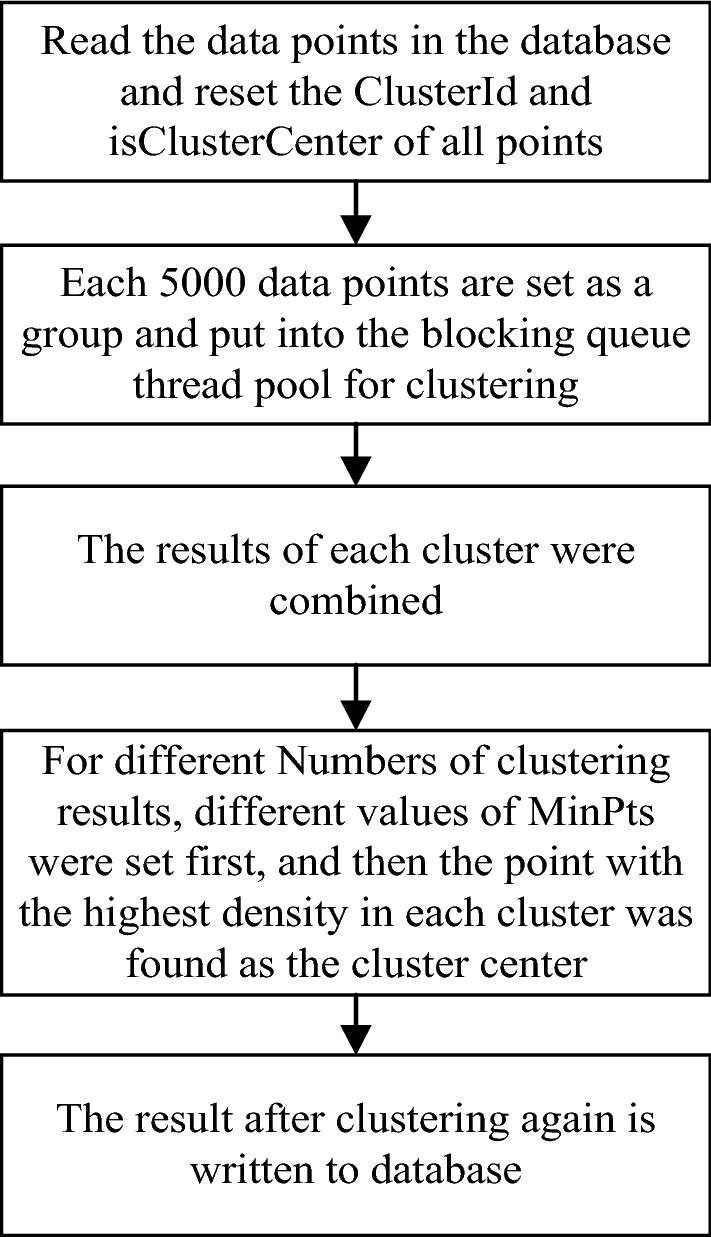
The block clustering flow chart.

The block clustering process is shown in Fig. 5. The DBSCAN^+^ algorithm aggregates data into a pool of blocked queue threads with a maximum thread count of 50 in a group of 5000. Thread pools are monitored during clustering. When the thread pool is empty, the clustering results are re-written to the *GPSList* of the trace point queue for re-clustering. Multiple experiments show that when *MinPts* is set to 2, more points will be counted in the result cluster. When *MinPts* is set to 4 or higher, the number of points in the result cluster will be too small, so the value of *MinPts* set in this paper is 3.

Based on prior knowledge, DBSCAN^+^ clustering method with 5000 data sets, *Eps* = 30 and *MinPts* = 3 as parameters was set for clustering. Empty the track point queue, and write the cluster center set back into the track point queue to cluster again and find the cluster center, until the number of cluster centers finally reaches the set number of passenger hot spots;

Set the output result points to less than 1000 to enhance the visual effect. In this algorithm, the *MinPts* minimum number is set for the problem that the selection of *MinPts* cannot simultaneously take into account the sparse remote areas and the dense passenger points in urban areas. When the number of *ClusterCenterList* (hereinafter referred to as *CCList*) of cluster centers meets the following conditions, the corresponding clustering parameters are set respectively: *CCList.Count* ≤ 1000, then the output requirements are met, and the clustering results can be written to the database directly. When *CCList.Count* > 1000 and *CCList.Count* ≤ 3000, the clustering was conducted with *MinPts* = 2 and *Eps* = 30. When *CCList.Count* > 3000, the clustering was conducted with *MinPts* = 3 and *Eps* = 30.

Finally, the clustering results are written to the database. When running the program again, all the points in each cluster are directly displayed, which saves the user’s time.

## Results

### Visualization method based on DBSCAN^+^

In the process of DBSCAN^+^ clustering, after the number of cluster centers *CCList.Count* satisfies the iterative termination condition, it traverses all the track points in the last track point queue, Through the cluster IDs of various clusters, the number of sample points in each cluster is counted separately, and the cluster aggregation degree Value corresponding to the cluster core is obtained. The ArcGIS 9.3 tool^28^ was used to generate the map. Based on the distribution density of the data, the tool dynamically renders the heat map of the point data by setting the search radius and weight field.

### Thermal visualization

**Figure 6:**
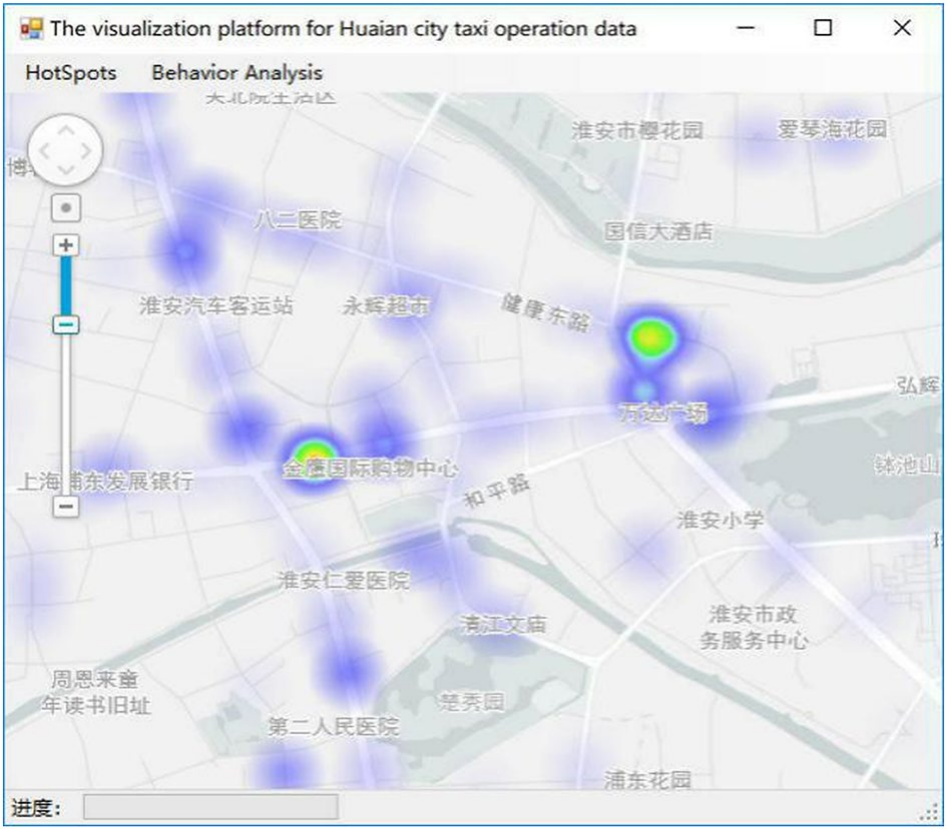
The original thermal diagram^28^.

The above-mentioned various cluster aggregation degree *Value* represents the heat of the passenger hotspot area in the visualization method. In thermal visualization, the color index of the highlighted area reflects the thermal power. The hotspots of each passenger area in the color highlight area are highlighted through setting the global maximum thermal value *MaxValue*, the thermal threshold ranges from 0 to *MaxValue* in this model. Because the data in the *Value* queue is not balanced, there may be a phenomenon that the maximum value is far beyond the multiple high-level aggregation areas. For example, if *MaxValue* is directly set to this value, the heat map effect cannot be scientifically presented, as shown in Fig. 6.

**Figure 7:**
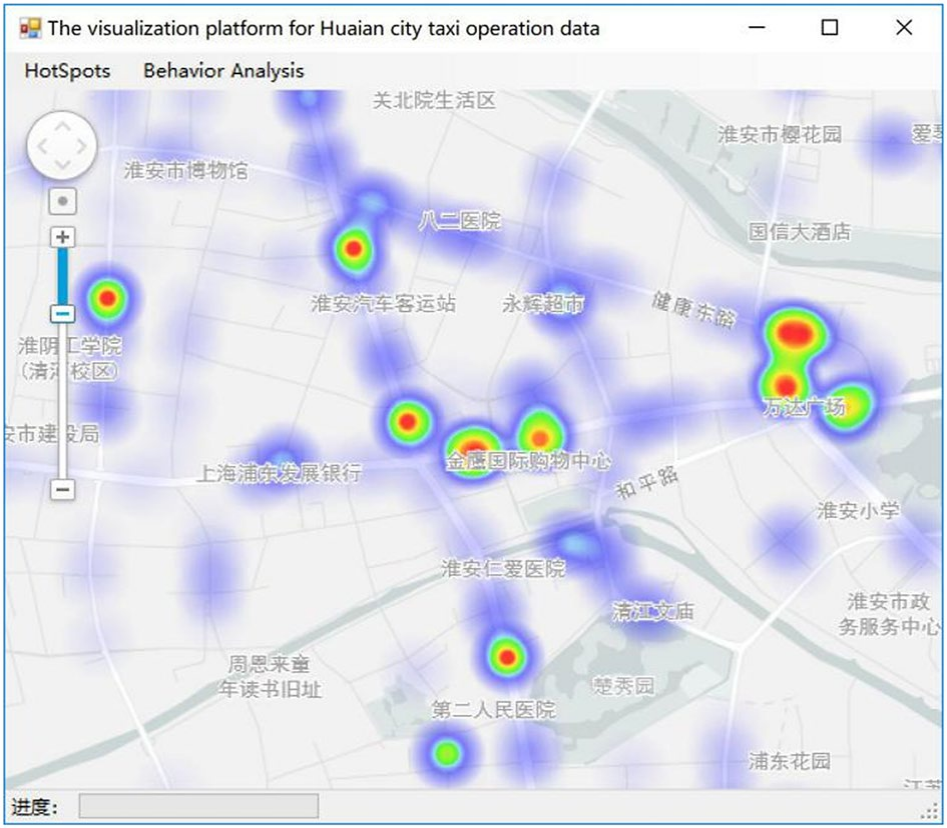
The optimized thermal diagram^28^.

Through K-Means++ algorithm, clustering the number of cluster sample points again, the maximum value are selected as the global maximum decision threshold of the heat map in the cluster center result, which can improve the generalization ability of the heat map model, correctly reflect multiple passenger hotspots in the city, and at the same time, even if the heat value of the low heat passenger area is low, the visualization effect is better. Here, the clustering effect is similar to filtering. By setting the parameters of each hot zone by combining various cluster cores and clusters of clusters, finally the heat map of the city is rendered to achieve the visual effect. The effect of thermal diagram is shown in Fig. 7.

### Visualization of address text

In order to facilitate the viewing of the study, the real geographical location of the passenger hotspot area is output. The cluster center is transformed from WGS-84 to GCJ-02 coordinate system^29^, and then from the cluster center set of GCJ-02 coordinate system to the corresponding real geographic location data set. Through POI (Point of Interest) reverse address analysis and combined with the thermal value of each passenger hot spot, the list of geographic information is output to Excel table. It can realize the transformation of data from latitude and longitude information to structured address information. for example: “lat: 33.600372, LNG: 119.045813” the result of inverse address analysis is “Wanda Plaza, 169 Xiangyu Middle Road, Qinjiangpu, Huai’an”. Exporting information can facilitate the relevant departments to make scheduling decisions and help drivers search for passengers faster in taxis.

## Discussion

### Analysis of experimental results

The experimental environment in this paper is a PC with Intel(R) Core(TM) i7-4700MQ CPU and 8 GB DDR3 1600 memory. Experimental data are from the taxi GPS data trajectory resources of Huai’an city from 2017 to 2018.

This paper experiments with the following density clustering algorithm for time complexity comparison. The original DBSCAN algorithm. TN-DBSCAN proposed by Wang Yafei^30^. GBA-DBSCAN proposed by Zhao^10^. Liu Chang et al.^31^ proposed Slice-DBSCAN by dividing the time slice. In most practical applications, machine learning systems must handle millions of users and billions of events. Therefore, as more and more users and events are added to the system, a well-designed data processing channel needs to be fast and scalable. This requires distributed computing. As far as our goal is concerned, Spark is a good choice as a distributed processing engine because it provides a computing framework that can execute many clustering tasks based on geolocation in parallel on multiple machines. Jindu et al.^32^ proposed a spatial data mining DBSCAN parallel clustering algorithm based on Spark platform.

Based on what the paper mentioned earlier, the density-based clustering algorithm parameters are set the same, the minimum scanning radius is set to 30 m, and the minimum number of cluster points is set to 3. Observe the performance comparison of different density clustering algorithms.

### Time complexity

The classic DBSCAN algorithm needs to traverse each sample point in the database during execution and perform an extended cluster operation on each sample point. In the extended cluster process, the neighborhood of the sample points needs to be scanned, so all points are traversed. The basic time complexity of the DBSCAN algorithm is *O*(*n*^2^).

The TN-DBSCAN algorithm uses the time dimension information in the clustering process to consider only the internal hotspots of the sliding window. Therefore, it reduces the number of original sample points to reduce the clustering time. The time complexity is still *O*(*n*^2^).

Based on GPS data, the improved GBA-DBSCAN in the clustering method for urban frequent congestion area identification, by dividing the urban area, defines the congestion frequency parameters for every other grid and accordingly identifies the frequently-occurring congestion area. The clustering problem is transformed into a grid, so that the original data set is reduced in dimension, thereby reducing the clustering time. Therefore, the time complexity of the algorithm itself is *O*(*n*^2^).

Based on the time slice division, Slice-DBSCAN reduces the number of data sets by taking snapshots of time segments and finds the congestion area by the density of taxis. The time complexity is the same as above.

The spatial data mining DBSCAN parallel clustering algorithm based on Spark platform is realized by parallel algorithm of single-node Spark platform, and then optimized from data transmission and serialization, and finally realizes cluster mode under Docker virtualization technology. Distributed clustering.

The DBSCAN^+^ algorithm adopts the scheme of first parallel clustering and then clustering the results again. Therefore, it can be understood that the local time complexity is *O*(*n*^2^). As the number of original sample points increases, the global time complexity becomes linear, so the time complexity is *O*(*n*). The DBSCAN^+^ algorithm partially uses the classic DBSCAN algorithm, so the data is divided into blocks and clustered in parallel. The local time complexity is *O*(*n*), and the overall time complexity is *O*(*n*^2^). It solves the problem of exponential increase in clustering time as the data increases.

Experiments show that the multi-threaded block-cycle clustering scheme reduces the time complexity of the algorithm from the exponential relationship of *O*(*n*^2^) to the linear relationship of *O*(*n*), which greatly shortens the clustering time of large-scale data. After several experiments, the average time is as shown in Table 2.

**Table 2:** Time performance of each density clustering algorithm.

Trajectory data volume	DBSCAN	TN-DBSCAN	GBA-DBSCAN	Slice-DBSCAN	Spark-DBSCAN	DBSCAN^+^
1000	1.235S	1.751S	1.678S	1.357S	3.125S	**1.478S**
1 W	4.124S	4.841S	4.735S	5.484S	7.865S	**4.145S**
10 W	397.815S	356.455S	342.716S	384.851S	32.157S	**42.354S**
100 W	∞	∞	∞	∞	268.164S	**432.214S**
1000 W	∞	∞	∞	∞	3481.412S	**5173.751S**

The bold indicates that the algorithm proposed in this paper is better than other algorithms.

### Clustering accuracy

In order to compare the clustering effects of different clustering algorithms, the DBI indicator was introduced. The Davies–Bouldin index (DBI), also known as the classification accuracy indicator, is an indicator proposed by David L. Davis and Donald Bouldin to evaluate the pros and cons of clustering algorithms. The DBI index is the ratio of the sum of the distances in the class to the distance outside the class and in order to evaluate the quality of the clustering. The goal is to ensure that the samples between each cluster are as close as possible and that the distance between samples of different clusters is as far as possible. Therefore, the smaller the DBI index, the better the clustering effect. The DBI index is calculated using the following formula:

8 $$ DBI = \frac{1}{N}\sum\limits_{i = 1}^{N} {\mathop {\max }\limits_{j \ne 1} } \left( {\frac{{\overline{S}_{i} + \overline{S}_{j} }}{{\left\| {w_{i} - w_{j} } \right\|_{2} }}} \right) $$

*S*_*i*_ calculates the average distance from the intra-class data to the centroid of the cluster, representing the degree of dispersion between the samples within the cluster. ||*w*_*i*_ -*w*_*j*_*||*_*2*_ represents the distance between the centroid of cluster class i and cluster class j. *N* represents the number of clusters.

The DBSCAN^+^ algorithm takes the centroid according to the principle of maximum density, which is different from the latitude and longitude of other algorithms. Block-wise parallel clustering results in a more even distribution of clusters than global clustering. Under the same environmental parameters, experiments show that DBSCAN^+^ algorithm clustering effect is superior to other similar clustering algorithms according to DBI index, which has advantages. The results are shown in Table 3. Marks et al.^33^ proposed Iterative DBSCAN (I-DBSCAN), which is an extension of the Density Based Spatial Clustering of Applications with Noise algorithm. POI-DBSCAN proposed by Mo^34^. In order to further evaluate the effectiveness of the algorithm, this paper and the above methods conducts experiments on the F-measure, Accuracy, and Purity indicators of the algorithm, the results are shown in Table 4.

**Table 3:** The DBI of each density clustering algorithm.

Trajectory data volume	DBSCAN	TN-DBSCAN	GBA-DBSCAN	Slice-DBSCAN	Spark-DBSCAN	DBSCAN +
1000	0.23421	0.24617	0.42234	0.37566	0.19842	**0.14897**
1 W	0.24512	0.29452	0.45219	0.41202	0.19522	**0.15242**
10 W	0.39215	0.29921	0.41756	0.38875	0.17561	**0.21435**

The bold indicates that the algorithm proposed in this paper is better than other algorithms.

**Table 4:** The external metrics of other indicator.

Method	Accuracy	Purity	F-measure
DBSCAN	0.734	0.692	0.1159
I-DBSCAN	0.788	0.751	0.1323
POI-DBSCAN	0.856	0.884	0.1352
DBSCAN^+^	**0.976**	**0.957**	**0.156**

The bold indicates that the algorithm proposed in this paper is better than other algorithms.

### Visual effect

**Figure 8:**
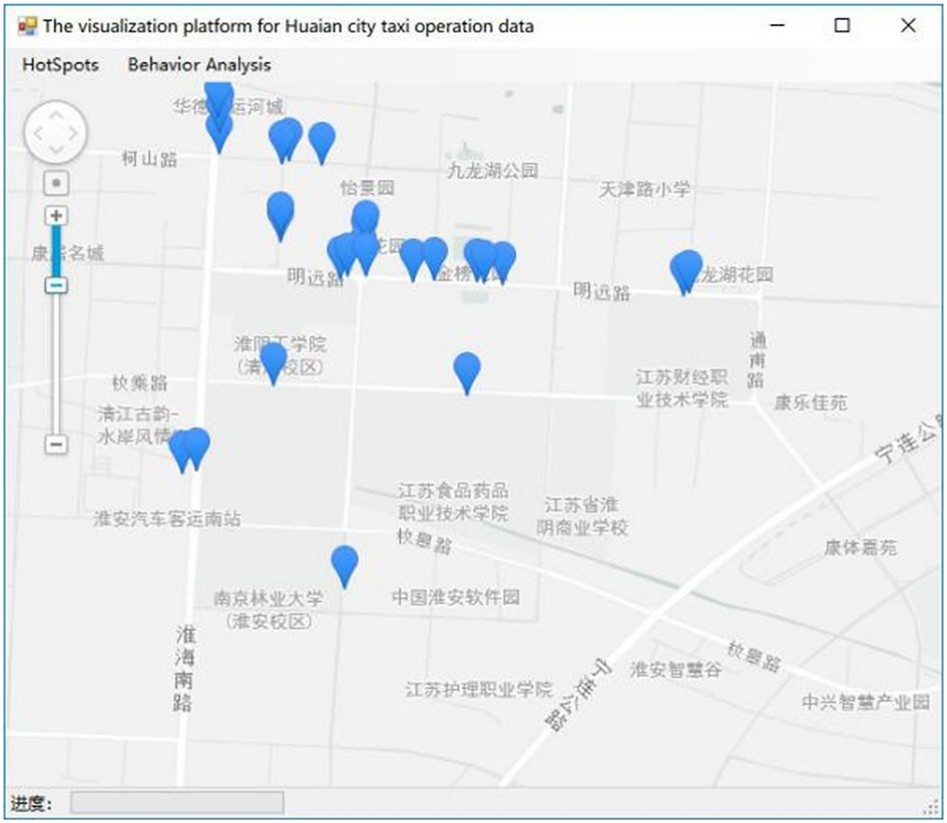
The diagram of passenger hotspot map^28^.

The result of the DBSCAN clustering algorithm is represented as a cluster of classes. Therefore, the hot spot shows an area on the map instead of the exact location. Even in hot spots, clustering results can cover the entire street. Using ArcGIS tool to import the data processed by DBSCAN^+^ algorithm and setting the centroid parameter, the effect is shown in Fig. 8. The visualization effect is more intuitive than the traditional algorithm, and it can specifically display the position represented by the centroid of the cluster.

**Figure 9:**
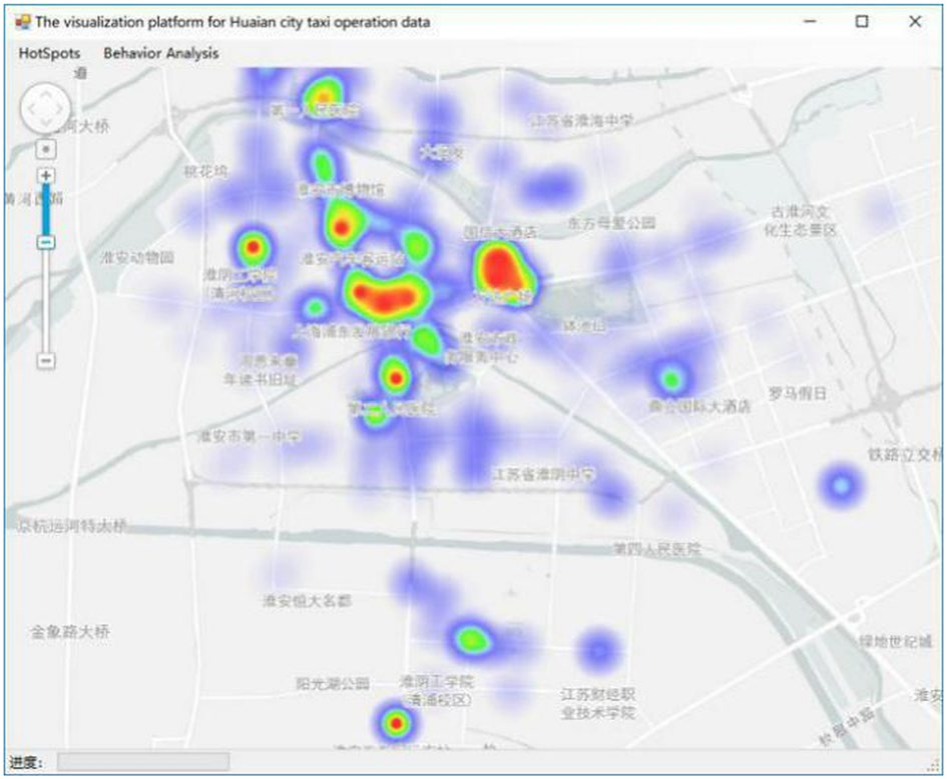
The diagram of thermal diagram visualization effect^28^.

Through the two visualized effects proposed in this paper based on DBSCAN^+^ algorithm, the heat carrying capacity of passengers in various regions of the city is presented intuitively and effectively. Combined with the thermal output precision of the passenger hot spot actual geographical location table, it overcomes the problem that the direct visualization of mass clustering hotspots on the map is not intuitive and is not easy to make decisions. In order to avoid the false death of the program caused by large data volume, slow network transmission, and other factors, the experiment tries to achieve high cohesion and low coupling of each module as far as possible. The threads of each module run independently and do not interfere with each other. Relevant ideas of software engineering were used to improve the availability and robustness of the programs. Selecting the symbol system in the ArcGIS tool, and performing heat map symbol rendering on the imported data. The effect is shown in Fig. 9. The DBSCAN^+^ algorithm is used to visualize the clustering heat map of Huai’an car-hire hot spots.

**Figure 10:**
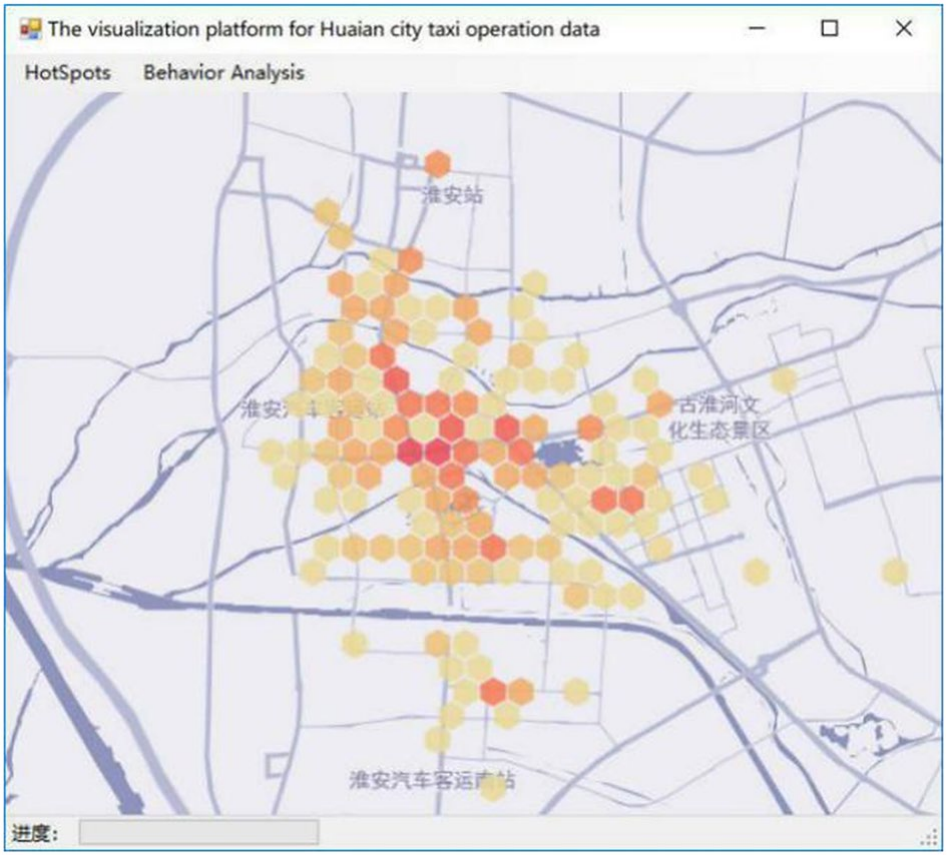
The diagram of Honeycomb heat diagram visualized effect^28^.

Similarly, different application scenarios are applied through the visualization of different thermal force models. Based on the clustering algorithm of DBSCAN^+^, the results of the passenger hot spots of a taxi in Huai’an city are shown through the honeycomb thermal diagram. Selecting the symbol system in the ArcGIS tool, and performing honeycomb map rendering on the imported data, and the results are shown in Fig. 10. Through the passenger hot spot density, the size of the customized area is set in the cellular heat map to facilitate the analysis of the number of business circles and administrative areas in the honeycomb with different passenger density, so as to be used in the decision-making of logistics optimization and precision marketing.

**Figure 11:**
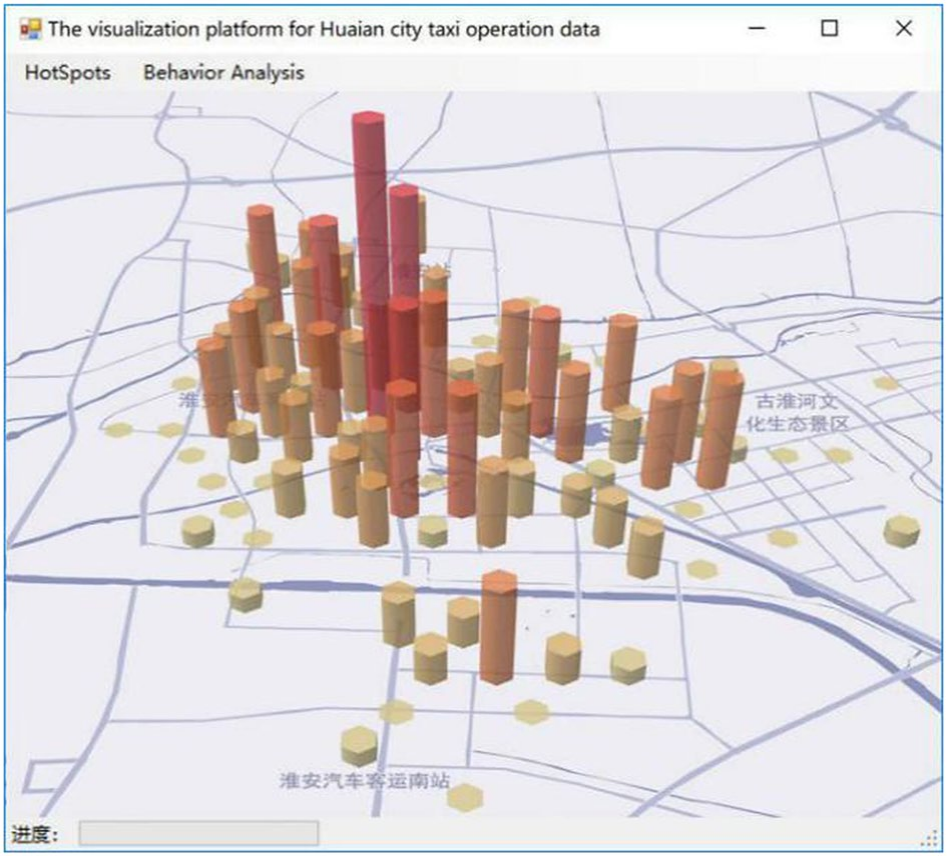
The visualization effect diagram of three-dimensional cylindrical honeycomb thermal diagram^28^.

Cylindrical three-dimensional honeycomb heat diagram shows the passenger density by the cylindrical height, which has a good illustration effect. The thermodynamic chart model is strengthened on the honeycomb thermodynamic chart, and the density color can be set to adjust the column radius, gap between columns and the maximum height of the column to show the passenger density in a three-dimensional manner, which also has all the advantages of the honeycomb thermal force. Selecting the symbol system in the ArcGIS tool, and performing histogram map rendering on the imported data, the visualization effect is shown in Fig. 11.

The experiment also can use POI reverse address resolution to show the detailed geographical location and aggregation degree output of Huai’an car-hire hot spot clustering based on DBSCAN^+^ algorithm. The physical location text output can be employed to quantify the passenger density in different areas from the data, thus providing data support for the corresponding research.

## Conclusions

In this paper, based on the GPS trajectory data of Huai’an city, DBSCAN^+^ algorithm was employed to cluster the passenger points extracted, so as to extract the passenger hot spots. In the visualization module, data is generated through algorithm analysis, and then generated on the open source map through the built-in tool of ARCGIS. Figure 6, 7, 8, 9, 10 and 11. were drawn by ArcGIS 9.3. It overcomes the traditional DBSCAN algorithm's inability to adapt to large-scale data, identify cluster centers, single-thread, and slow clustering speed. The DBSCAN^+^ algorithm is compared with DBSCAN and GBADBSCAN algorithm in Huai’an taxi GPS trajectory. Experimental proof DBSCAN^+^ algorithm in time complexity, resource utilization, the clustering accuracy, and visual effect has certain advantages, accurate extraction of taxi passenger hot spots has a better effect and it also can better reflect the road passenger fever. At the same time, the visualization effect proposed based on DBSCAN^+^ algorithm overcomes the problem that the number of passengers cannot be displayed directly on the map due to the large number and density of passenger hot spots.

This method can directly and effectively display the heat capacity of each region of the city and output the accurate geographical position table of the hot spot after the heat value is output. This will make it easier for relevant departments to make decisions on taxi operation and scheduling and help drivers find passengers waiting in hot spots more quickly.

DBSCAN^+^ algorithm improves clustering efficiency by partitioning data, which can greatly reduce the time complexity. The algorithm emphasizes local clustering but ignores the relationship between the data as a whole. At the same time, in different densities, the algorithm has not yet realized the automatic optimization of parameters, and the number of clustering results cannot be accurately controlled. This is the direction of improvement in the next step.

## Data Availability

We can provide the data.

## References

[1] Verma, N. & Baliyan, N. PAM clustering based taxi hotspot detection for informed driving. In *IEEE International Conference on Computing, Communication and Networking Technologies (ICCCNT)* 1–7 (2017).

[2] Bischoff, J., Maciejewski, M. & Sohr, A. Analysis of Berlin's taxi services by exploring GPS traces. In *IEEE International Conference on Models and Technologies for Intelligent Transportation Systems* 209–215 (2015).

[3] Phiboonbanakit, T. & Horanont, T. How does taxi driver behavior impact their profit? Discerning the real driving from large scale GPS traces. In *ACM International Joint Conference on Pervasive and Ubiquitous Computing: Adjunct* 1390–1398 (2016).

[4] Hoque, M. A., Hong, X. & Dixon, B. Analysis of mobility patterns for urban taxi cabs. In *IEEE International Conference on Computing* 756–760 (2012).

[5] Luo Y, Ding T, Zhu M (2017). Road2vec: a visual analysis method of urban road with taxi trajectory data. J. Comput. Aided Des. Comput. Graph..

[6] Wan, X. J., Wang, J., Du, Y., *et al.* DBH-CLUS: a hierarchic clustering method to identify pick-up/drop-off hotspots. In *International Conference on Intelligent Computing* 330–341 (Springer, 2015).

[7] Zheng, L., Xia, D., Zhao, X., *et al.* Mining trip attractive areas using large-scale taxi trajectory data. In *Ubiquitous Computing and Communications (ISPA/IUCC), 2017 IEEE International Symposium on Parallel and Distributed Processing with Applications* 1217–1222 (2017).

[8] Fan, K., Zhang, D., Wang, Y., *et al.* Discovering urban social functional regions using taxi trajectories. In *IEEE International Conference on Autonomic and Trusted Computing* 356–359 (2016).

[9] Kumar KM, Reddy ARM (2016). A fast DBSCAN clustering algorithm by accelerating neighbor searching using groups method. Pattern Recognit..

[10] Zhao L (2014). Loading Situation Visual Analysis based on Taxi Trajectory Data.

[11] Feng Q (2017). Research on Residents’ Trip Hot Routes and Attractive Areas Based on Taxi Trajectory Data.

[12] Zhao P (2015). Research on the Method of Extracting and Analyzing Urban Hotspots Based on Trajectory Clustering.

[13] Jiang H, Yu Y (2017). Extracting fine pickup hot zone by an improved DBSCAN algorithm. Geospat. Inf..

[14] Zheng Y, Zhao G, Liu J (2016). A novel method for traffic hotspots recognition based on taxi GPS data. J. Beijing Inf. Sci. Technol. Univ..

[15] Qing Z, Kun Q, Yi-Xiang C (2016). Hotspots detection from taxi trajectory data based on data field clustering. Geography Geo-Inf. Sci..

[16] Kong X, Liu Y, Wang Y (2017). Investigating public facility characteristics from a spatial interaction perspective: a case study of Beijing hospitals using taxi data. ISPRS Int. J. Geo-Inf..

[17] Wang, R., Chow, C. Y., Yan, L., *et al.* TaxiRec: recommending road clusters to taxi drivers using ranking-based extreme learning machines. In *ACM International Conference on Advances in Geographic Information Systems* 1–4 (2015).

[18] Liu, D., Cheng, S. & Yang, Y. Density peaks clustering approach for discovering demand hot spots in city-scale taxi fleet dataset. In *IEEE International Conference on Intelligent Transportation Systems* 1831–1836 (2015).

[19] Yun, S., Sang, H., Ju, S., *et al.* Taxi cab service optimization using spatio-temporal implementation to hot-spot analysis with taxi trajectories: a case study in Seoul, Korea. In *ACM Sigspatial International Workshop on Mobile Geographic Information Systems* 12–18 (2016).

[20] Chen C, Zhang D, Li N (2014). B-Planner: planning bidirectional night bus routes using large-scale taxi GPS traces. IEEE Trans. Intell. Transp. Syst..

[21] Lee, J., Shin, I. & Park, G. L. Analysis of the passenger pick-up pattern for taxi location recommendation. In *IEEE International Conference on Networked Computing and Advanced Information Management* 199–204 (2008).

[22] Xiao, L., Fan, X., Mao, H., *et al.* When taxi meets bus: night bus stop planning over large-scale traffic data. In *IEEE International Conference on Cloud Computing and Big Data* 19–24 (2017).

[23] Zhang D, Sun L, Li B (2015). Understanding taxi service strategies from taxi GPS traces. IEEE Trans. Intell. Transp. Syst..

[24] Mazimpaka, J. D. & Timpf, S. Exploring the potential of combining taxi GPS and flickr data for discovering functional regions. In *AGILE 2015* 3–18 (Springer, Cham, 2015).

[25] Zhou Z, Dou W, Jia G (2016). A method for real-time trajectory monitoring to improve taxi service using GPS big data. Inf. Manag..

[26] Wang Y, Yang W, Xu Z (2017). Excavation of passenger hotspots based on taxi trajectory. China Comput. Commun..

[27] Kumar D, Wu H, Rajasegarar S (2018). Fast and scalable big data trajectory clustering for understanding urban mobility. IEEE Trans. Intell. Transp. Syst..

[28] The [Figure6–Figure11] for this paper was generated using [ArcGIS] platform, Version [9.3] for developers. Copyright © 2020 Esri. RedLands, California, USA. Available at: https://developers.arcgis.com/.

[29] Wang Z, Lu M, Yuan X (2013). Visual traffic jam analysis based on trajectory data. IEEE Trans. Vis. Comput. Graph..

[30] Wang YF (2014). Visual Analysis of Passenger Status Based on Taxi Trajectory Data.

[31] Liu Ch, Li ZJ, Jiang SX (2015). Discovery of urban traffic congestion areas based on DBSCAN algorithms. Intell. Comput. Appl..

[32] Jin D (2017). Parallelization of DBSCAN Clustering Algorithms for Spatial Data Mining Based on Spark Platform.

[33] Marks, C., Jahangiri, A. & Machiani, S. G. Iterative DBSCAN (I-DBSCAN) to identify aggressive driving behaviors within unlabeled real-world driving data. In *2019 IEEE Intelligent Transportation Systems Conference (ITSC)* 2324–2329 (2019).

[34] Mo, F. & Yamana, H. Point of interest recommendation by exploiting geographical weighted center and categorical preference. In *2019 International Conference on Data Mining Workshops (ICDMW)* 73–76 (2019).

